# Epilepsy surgery, vision, and driving: What has surgery taught us and could modern imaging reduce the risk of visual deficits?

**DOI:** 10.1111/epi.12372

**Published:** 2013-09-20

**Authors:** Gavin P Winston

**Affiliations:** Epilepsy Society MRI Unit, Department of Clinical and Experimental Epilepsy, UCL Institute of NeurologyLondon, United Kingdom

**Keywords:** Temporal lobe epilepsy, Visual field deficit, Anterior temporal lobe resection, Selective amygdalo-hippocampectomy, Diffusion tensor imaging

## Abstract

Up to 40% of patients with temporal lobe epilepsy (TLE) are refractory to medication. Surgery is an effective treatment but may cause new neurologic deficits including visual field deficits (VFDs). The ability to drive after surgery is a key goal, but a postoperative VFD precludes driving in 4–50% of patients even if seizure-free. VFDs are a consequence of damage to the most anterior portion of the optic radiation, Meyer's loop. Anatomic dissection reveals that the anterior extent of Meyer's loop is highly variable and may clothe the temporal horn, a key landmark entered during temporal lobe epilepsy surgery. Experience from surgery since the 1940s has shown that VFDs are common (48–100%) and that the degree of resection affects the frequency or severity of the deficit. The pseudowedge shape of the deficit has led to a revised retinotopic model of the organization of the optic radiation. Evidence suggests that the left optic radiation is more anterior and thus at greater risk. Alternative surgical approaches, such as selective amygdalo-hippocampectomy, may reduce this risk, but evidence is conflicting or lacking. The optic radiation can be delineated in vivo using diffusion tensor imaging tractography, which has been shown to be useful in predicting the postoperative VFDs and in surgical planning. These data are now being used for surgical guidance with the aim of reducing the severity of VFDs. Compensation for brain shift occurring during surgery can be performed using intraoperative magnetic resonance imaging (MRI), but the additional utility of this expensive technique remains unproven.

Epilepsy is one of the most common and serious neurologic disorders (Sander & Shorvon, 1996). Up to 40% of patients with temporal lobe epilepsy (TLE) are refractory to medication (Semah & Ryvlin, 2005). Temporal lobe surgery is an established and effective treatment (Wiebe et al., 2001), but the benefits of surgery must be weighed against the possible adverse neurologic, psychological, and psychiatric consequences.

Meyer's loop, the most anterior portion of the optic radiation, passes through the temporal lobe, so is at risk during surgery. The ability to drive is a key goal of patients who are undergoing surgery (Taylor et al., 2001), but postoperative visual field deficits (VFDs) are significant enough to preclude driving in between 4% and 50% of patients, even if seizure-free (Manji & Plant, 2000; Pathak-Ray et al., 2002; Jeelani et al., 2010).

Anatomic dissection and experience from temporal lobe surgery since the 1940s have provided copious information on the anatomy of the optic radiation and the consequences of surgery. More recently, diffusion tensor imaging tractography has enabled depiction of the optic radiation in vivo, with these data now routinely used to guide tumor neurosurgery. These data are being increasingly used for epilepsy surgery and hold promise to reduce the risk of postoperative VFDs.

In this review, I discuss the anatomy of the optic radiation, the visual outcomes following temporal lobe epilepsy surgery using data from the early pioneers to the present day, and how tractography data have been used and could be used in the future to assist surgery and increase the proportion of patients able to drive.

## Anatomy of the Optic Radiation

**Figure 1:**
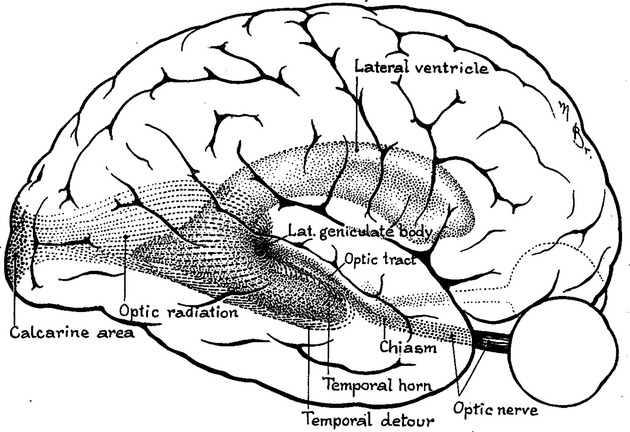
Optic radiation viewed from lateral aspect showing the “temporal detour” (Meyer's loop). Derived from Cushing's original drawing in 1921 (Cushing, 1921).

The optic radiation is the final part of the visual pathway connecting the lateral geniculate nucleus (LGN) of the thalamus to primary visual cortex in the occipital lobe (Fig. 1). Following decussation in the optic chiasm, each optic radiation conveys the contralateral visual field from both eyes. Adolf Meyer used dissection to describe three bundles of the optic radiation and the “peculiar detour of the ventral portion of the geniculocalcarine path [optic radiation]” (Meyer, 1907):

1. Anterior bundle (ventral) passing anteriorly over the roof of the temporal horn of the lateral ventricle and then turning backward (Meyer's loop) along the inferolateral aspect of the temporal horn before turning to run underneath the ventricle and passing into the lower lip of the calcarine fissure.
2. Central bundle (lateral) passing laterally across the roof of the temporal horn and then posteriorly along the lateral wall and roof of the trigone and occipital horn to the occipital pole.
3. Posterior bundle (dorsal) passing directly posteriorly along the roof of the trigone and occipital horn, radiating into the upper lip of the calcarine fissure.

The anterior bundle represents the superior visual quadrant, the posterior bundle the inferior quadrant, and the central bundle conveys macula vision (Meyer, 1907; Harrington, 1961). Support for this division came from Gordon Holmes' studies of VFD from gunshot wounds in World War I (Holmes, 1918), Harvey Cushing's series of patients with temporal lobe tumors (Cushing, 1921), and patients with penetrating head injuries (Spalding, 1952). However, these early studies did not address the anterior extent of Meyer's loop or the organization of fibers within the optic radiation and thus the nature and congruity of any deficit. The advent of temporal lobe epilepsy surgery began to address these and other questions.

## Temporal Lobe Epilepsy Surgery

Anterior temporal lobe resection (ATLR) for epilepsy was pioneered by Wilder Penfield from the Montreal Neurological Institute and Murray Falconer from the Guy's-Maudsley Neurosurgical Unit, London from the late 1940s onward. It was recognized that surgery often resulted in VFD, providing an ideal opportunity to study vision with a well-defined and reproducible insult. Penfield observed:

When the line of removal is less than 6 cm posterior to the tip, it is apt to result in no postoperative visual field defect. If the removal includes 6 cm and more, it is apt to produce contralateral upper quadrantic homonymous hemianopia. When the line is pushed back to 8 cm, there is apt to be a complete homonymous hemianopic defect. (Penfield, 1954)

However, Falconer noted intersubject variability because a 6-cm resection caused hemianopia in one patient, but an 8-cm resection resulted in virtually no visual field loss in another (Falconer et al., 1955). Many subsequent studies have been published that have further refined this risk (Table 1) and from which the following conclusions can be drawn.

**Table 1:** Literature on VFDs following ATLR and SAH

Authors (year), center (surgery years)	Surgical technique (resection size)	Number	VF	% with VFD and size of postoperative deficit	Relationship of VFD to resection size	Proposed anterior limit of Meyer's loop
Bjork and Kugelberg (1957) Stockholm	Temporal lobectomy (4–6.5 cm)	26	G ± B	25/26 (96%) <q	Yes, greater loss with larger resection	30–40 mm, anterior to temporal horn
Falconer and Wilson (1958) Guy's-Maudsley	Temporal lobectomy (4.5–9 cm)	50	G + B or C	50/50 (100%) <q/q/>q/h	Variable, >q more likely if 8–9 cm	<45 mm, no comment on temporal horn
Van Buren and Baldwin (1958) NINDS, NIH, U.S.A.	Temporal lobectomy (unknown)	44	G + B	33/41 (80%) <q/q	–	Posterior to temporal horn
Wendland and Nerenberg (1960) Minnesota (1952–1960)	Temporal lobectomy (5–10 cm)	24	a	24/24 (100%) [9<q, 7q, 2>q, 6h]	Yes, related but marked variability	–
French (1962) Minnesota (1948–1961)	Temporal lobectomy (5–9 cm)	30	a	30/30 (100%) [13<q, 9q, 2>q, 6h]	Yes, greater loss with larger resection	–
Marino and Rasmussen (1968) Montreal (1962–1967)	Temporal lobectomy (4–8 cm)	50	b	33/50 (66%) [26<q, 3q, 3>q, 1 h]	Yes, related but marked variability	<40 mm, posterior to temporal horn
Jensen and Seedorff (1976) Copenhagen (1960–1969)	Temporal lobectomy (5.5–7 cm; 6 cm D, 7 cm ND)	74	B	51/69 (74%) [38<q/q, 7>q, 6h]	No, but larger VFD in R-sided resection (larger)	May/may not involve temporal horn
Babb et al. (1982) Los Angeles, UCLA	Temporal lobectomy (5–7.5 cm)	22	G	13/22 (59%) [3<q, 7q/>q, 3 h]	No, but correlates with parahippocampal VEP	–
Spencer et al. (1984) Yale	Standard temporal lobectomy (6.0–6.5 cm ND, less by ECoG D)	17	–	14/15 (93%) [1<q, 9q, 4>q]	–	–
	Modified temporal lobectomy (4.5 cm, reduced 3 cm in D STG)	19	–	15/16 (94%) [5<q, 9q, 1>q]	–	–
Wieser (1986) Zurich (1983–)	SAH (Yasargil)	13	O	0/13 (0%)	–	–
Katz et al. (1989) Cleveland	Temporal lobectomy (unknown)	45	G	27/39 (69%) <q/q	No, but resections larger in those with VFD (MRI)	–
Tecoma et al. (1993) San Francisco (1986–1991)	Temporal lobectomy (3.5–5 cm D, 4–6.5 cm ND)	33	G	17/33 (52%)	–	–
Renowden et al. (1995) Oxford (1989–1992)	Transcortical SAH (Niemeyer)	7	–	4/7 (57%) <q	–	–
	Transsylvian SAH (Yasargil)	10		5/10 (50%) <q		
Vajkoczy et al. (1998) Munich/Heidelberg (1990–1996)	Transsylvian-transcisternal SAH	32	G	1/32 (3%) q	–	–
Hughes et al. (1999) Vanderbilt (1990–1995)	Temporal lobectomy (4–7 cm)	32	H	31/32 (97%)	Yes, contralateral deficit worse in >6 vs. <5 cm	–
Manji and Plant (2000) Queen Square (1986–1995)	Temporal lobectomy (unknown)	24	C	5/24 (21%)	–	–
			G	13/24 (54%) [10/24 (42%) fail DVLA]		
			E	11/24 (46%) [6/24 (25%) fail DVLA]		
Krolak-Salmon et al. (2000) Lyon (1994–1998)	Temporal lobectomy (2–6 cm D, 2–7 cm ND)	18	M	15/18 (83%) <q/q	Yes, more likely with larger resection (MRI)	20–30 mm, anterior to temporal horn
Egan et al. (2000) Portland, U.S.A.	Modified temporal lobectomy (3.5–4 cm)	15	G	11/15 (73%) <q	–	–
	Transcortical SAH (Niemeyer)	14		11/14 (79%) <q	–	–
Hervas-Navidad et al. (2002) Granada, Spain (1995–1998)	Temporal lobectomy (unknown)	30	H	27/30 (90%) [15<q, 3q, 8>q, 1 h]	Yes, greater loss with larger resection (MRI), variable	–
Pathak-Ray et al. (2002) Cardiff (10 years)	Temporal lobectomy (unknown)	14	H	9/14 (64%) [8<q, 1q, 2 vigabatrin]	–	–
			E	7/14 (50%) failed DVLA [4<q, 1q, 2 vigabatrin]		
Nilsson et al. (2004) Gothenburg (1987–1999)	Standard temporal lobectomy (5–5.5 cm D, 6 cm ND)	33	G	16/33 (48%) [9<q, 6q, 1 h]	Yes, correlated with anterior STG resection (MRI)	Involvement of STG at 18–36 mm
	Modified temporal lobectomy (3 cm D + less of STG, 3.5–4.5 cm ND)	17		9/17 (53%) [5<q, 4q]		
Barton et al. (2005) Boston (9 months)	Temporal lobectomy	29	G	29/29 (100%)	Yes, linear regression with size of resection (MRI)	24 mm nasal, 28 mm temporal, 32 mm temporal horn
Yeni et al. (2008) Istanbul	Transsylvian SAH (Yasargil)	30	H	11/30 (37%) <q/q	–	–
Mengesha et al. (2009) Vanderbilt (2001–2006)	Transcortical SAH (Niemeyer)	18	H	16/18 (89%) [13<q/q, 3>q]	–	–
Mengesha et al. (2009) Vanderbilt (1990–1995)	Standard temporal lobectomy (4–7 cm)	33	H	30/33 (91%) [<q/q]	–	–
Jeelani et al. (2010) Queen Square (1984–2004)	Modified temporal lobectomy (4–4.5 cm)	105	E	16/105 (15%) [4/105 (4%) fail DVLA]	No, but resections all stereotyped at 4–4.5 cm (MRI)	<45 mm, but cannot assess further

*Surgical technique*: D, dominant; ND, nondominant; ECoG, electrocorticography. *Visual field technique* (*VF*): B, Bjerrum campimetry; C, confrontation; E, Esterman (binocular); G, Goldmann; H, Haimark; M, Metrovision; O, Octopus. *Visual field deficits* (*VFDs*): <q, partial quadrantanopia; q, complete quadrantanopia; >q, greater than quadrantanopia; h, hemianopia; DVLA, Driving and Vehicle Licensing Agency guidelines for driving (United Kingdom).

a Mixture of central fields + perimetry (unspecified).

b Haimark perimetry + tangent.

### Type of perimetry

Early studies tended to use manual kinetic perimetry (e.g., Goldmann, Haimark), which allows a detailed mapping of the visual fields. It is, however, affected by subject and observer variability. More recent studies use automated static perimetry, either monocular (Humphrey; Metrovision) or binocular (Esterman). These are more reproducible and easier to assess quantitatively, but the binocular Esterman test has a low sensitivity for VFDs (Manji & Plant, 2000).

### Frequency of deficits

VFDs following temporal lobe surgery are common (48–100%). The single outlying study showing a 15% risk is likely to be related to the use of binocular Esterman perimetry and bias by only counting “significant” VFDs (Jeelani et al., 2010).

### VFD and resection size

Studies show a relationship between the size of resection and either the risk of, or severity of, VFD. A key finding is substantial variability between subjects, reflecting anatomic variability in the location of Meyer's loop. The original 6-cm safety margin proposed by Penfield (1954) is clearly an overestimate, with subsequent studies suggesting margins of 30–40 mm (Bjork & Kugelberg, 1957) and 45 mm (Falconer & Wilson, 1958) from the temporal pole to avoid a VFD. Over time, estimates have gradually reduced further. Despite the anatomic variability, a linear relationship between the severity of a VFD and the degree of resection (anteroposterior distance on postoperative magnetic resonance imaging [MRI]) has been shown (Barton et al., 2005). This model predicts that the ipsilateral nasal field is on average involved with resections ≥24 mm or the temporal pole, and the contralateral temporal field in resections ≥28 mm or the temporal pole.

### Relationship of Meyer's loop to the temporal horn

Cushing (1921) and Bjork (Bjork & Kugelberg, 1957) suggest that the optic radiation clothes the temporal horn and lies anterior to it. However, Van Buren (Van Buren & Baldwin, 1958) and Marino (Marino & Rasmussen, 1968) depict the optic radiation ending just posterior to the temporal horn. Recent studies suggest that the optic radiation is anterior to the temporal horn, at least in the majority of cases. In one study, the average distance from temporal pole to temporal horn was 32 mm, with the optic radiation predicted to be 24 mm on average from the temporal pole (Barton et al., 2005), which is supported by dissection studies. This is of importance as the temporal horn is a key landmark entered during the standard neurosurgical approach for ATLR.

### Nature of the VFD

The medial border of the deficit is generally sharp and superimposed on the vertical meridian, whereas the inferior border slopes toward the point of fixation and the isopters are separated. Early papers describe a “wedge”-shaped deficit, and some refer to the inferior border being radial. Close observation of the published field deficits, however, suggests that the inferior border although sloping does not point toward the central point of fixation, and that the deficits are more of a “pseudowedge.”

No consensus on the congruity of deficits exists. Some studies report that most or all of deficits are congruous, whereas others report incongruous deficits that are typically larger on the ipsilateral side. Barton found a consistent 15% greater VFD on the ipsilateral side regardless of the extent of resection and laterality (Barton et al., 2005). Many authors have used this as evidence that fibers from the ipsilateral eye are more laterally located (Van Buren & Baldwin, 1958; Hughes et al., 1999).

Similar disagreement exists as to the degree of macular involvement. The majority of studies report macula involvement only for VFD greater than a quadrantanopia (Falconer & Wilson, 1958; Marino & Rasmussen, 1968; Jensen & Seedorff, 1976; Babb et al., 1982) or macular sparing in studies where only partial quadrantanopsias are seen (Bjork & Kugelberg, 1957; Egan et al., 2000). However, some studies do suggest that the macula may be involved in significant quadrantanopsias (Hughes et al., 1999; Krolak-Salmon et al., 2000; Barton et al., 2005).

### Retinotopic organization of the optic radiation

Van Buren suggested a retinotopic organisation in which the visual field adjacent to the vertical meridian is represented most anteriorly in the optic radiation, with radial degrees from vertical to horizontal represented going more posteriorly (Fig. S1; Van Buren & Baldwin, 1958). This was used to explain the range of deficits from the small paravertical deficits, through wedge-shaped deficits with a sloping inferior border to a complete quadrantanopia. This converts rapidly to a hemianopia with larger resections, with deficits between a quadrantanopia and hemianopia seen only rarely.

Barton suggests a revised model in which the superior field (rather than that by the vertical meridian) is represented most anteriorly, with more inferior portions being represented more posteriorly (Barton et al., 2005). Furthermore, the macula is represented most posteriorly and ipsilateral fibers are slightly more anterior than the contralateral fibers (rather than more lateral as previously suggested). This 90-degree rotation coupled with the principle of central magnification better explains the observed pseudowedge deficits and could explain the linear relationship seen between resection size and severity of VFDs.

### Left/right asymmetry of VFDs

An early study showed the frequency of VFDs was similar for left- and right-sided resections but that the severity of the VFD was greater in right-sided resections (Jensen & Seedorff, 1976). This was postulated to be a result of larger resections in the nondominant hemisphere. Although the findings were replicated in one study (Hervas-Navidad et al., 2002), another found that right-sided resections did not lead to greater VFD despite larger resections (Hughes et al., 1999). Furthermore, in a study that assessed resection size with postoperative MRI, the average VFD did not differ between left- and right-sided resections for any given size of resection (Barton et al., 2005).

There is mounting recent evidence, however, that the left optic radiation may be more anterior. Data from hippocampal visual evoked potentials suggest that the anterior extent of the optic radiations may differ by as much as 1.5 cm (Babb et al., 1982). In a cohort undergoing selective amygdalo-hippocampectomy, VFDs were seen in 10/21 (left-sided resection) but only 1/9 (right-sided resection; Yeni et al., 2008). Furthermore, in a large cohort of patients undergoing ATLR the odds ratio for a VFD was 3.51 for a left-sided versus a right-sided resection, with no significant difference in the extent of resection between the two sides (Jeelani et al., 2010).

### Relevance of the deficit to driving

Although patients are typically unaware of the deficit, intact vision is essential for a driving licence, one of the key aims for patients having epilepsy surgery (Taylor et al., 2001). Current European guidelines (European Commission Directive 2009/112/EC, available online at http://eur-lex.europa.eu/LexUriServ/LexUriServ.do?uri=OJ:L:2009:223:0026:0030:EN:PDF.) require “the horizontal visual field should be at least 120 degrees, the extension should be at least 50 degrees left and right and 20 degrees up and down. No defects should be present within a radius of the central 20 degrees.” In the United Kingdom, this is assessed by binocular Esterman perimetry (Driver & Vehicle Licensing Agency, 2013).

Relatively few studies have looked at vision and driving following epilepsy surgery. In 24 patients who had undergone ATLR, 13 had a deficit detected by Goldmann perimetry, with 10 failing to meet driving criteria (42%), but using the more lenient Esterman test, deficits were shown in only 11, with 6 failing driving criteria (25%; Manji & Plant, 2000). In a study in a more homogenous group undergoing ATLR for hippocampal sclerosis alone, 7 (50%) of 14 failed Driver and Vehicle Licensing Agency (DVLA) criteria (Pathak-Ray et al., 2002) but two patients had a preexisting VFD from vigabatrin use. The most recent study suggested that only 4% of a consecutive series of 105 patients failed to meet driving criteria (Jeelani et al., 2010).

The reasons for the discrepant figures are unclear, but may be partly related to different surgical approaches. These are all, however, historical series in patients with predominantly hippocampal sclerosis. Improved imaging techniques have enabled surgery in patients where it was not previously possible, including neocortical or nonlesional epilepsy (Duncan, 2010), where the risk to vision may be greater if the epileptogenic cortex is located more posteriorly and thus overlying more of the optic radiation.

### Effect of the surgical technique

The surgical technique has changed over the years in two major ways—the modification of the technique for temporal lobectomy, and the introduction of selective amygdalo-hippocampectomy (SAH).

The standard ATLR, comprising en bloc resection of both medial temporal (amygdala, anterior hippocampus) and lateral temporal neocortical structures, was described by Falconer from the Maudsley Hospital (Falconer et al., 1955), and Morris (1956) stated that it should include 6.5 cm of lateral temporal cortex, the uncus, the amygdala, and 2–4 cm of anterior hippocampus. However, such extensive resections are prone to postoperative neuropsychological and visual deficits.

In the late 1970s, modifications to the standard procedure were made. Spencer described a technique whereby removal of the medial structures could be achieved with only a more limited temporal pole resection, thereby preserving the remaining lateral temporal neocortex (Spencer et al., 1984). Resections were limited to 4.5 cm of the superior, middle, and inferior temporal gyri in the nondominant hemisphere, with a further reduction to 3 cm of the superior temporal gyrus in the dominant hemisphere. The two studies, which have compared the visual outcome in standard and modified temporal lobectomy (Spencer et al., 1984; Nilsson et al., 2004), both found that although the frequency of a VFD was unchanged, the VFDs were less severe.

The second approach is to perform a more selective resection of mesial structures with preservation of the lateral temporal neocortex (Wieser & Yasargil, 1982). A variety of surgical approaches to the temporal lobe exist for this procedure, which can divided into three groups (Sincoff et al., 2004), but large studies of visual outcomes are lacking.

The transcortical-transventricular SAH introduced by Niemeyer (1958) enters via the middle temporal gyrus and puts the optic radiation at risk, as the lateral aspect of the temporal horn is deep to the superior and middle temporal gyri. Two studies comparing ATLR and transcortical SAH reveal that the frequency of VFD does not differ between these operations (Egan et al., 2000; Mengesha et al., 2009), although the lateral part of vision is less severely affected (Mengesha et al., 2009).

Subtemporal approaches should avoid the optic radiation as they do not involve the floor of the temporal horn, but do risk damage to the vein of Labbe by retraction. Entry via the fusiform gyrus did not cause a VFD in four patients (Hori et al., 1993), whereas entry via the parahippocampal gyrus resulted in a single case of quadrantanopia in seven patients (Park et al., 1996).

Finally, Yasargil describes a transsylvian-transventricular approach, with access to the temporal horn via an incision in the superior temporal gyrus at the level of limen. Avoiding the lateral temporal cortex makes this a technically demanding operation, and entry via the temporal stem passing through the uncinate fasciculus may put the optic radiation at risk. This operation has a very low risk of VFDs in the hands of the inventor (2 of 173 patients affected; Yasargil et al., 2004), but this success has not been reproduced by other surgeons applying this technique (Renowden et al., 1995; Yeni et al., 2008).

Overall, although there is a suggestion that alternative surgical approaches may reduce the risk or severity of a VFD, these findings are not always replicated and need validation in larger series.

## Anatomic Dissection Studies

The introduction of the Klingler's fiber dissection technique (Ludwig & Klingler, 1956) allowed careful study of the location and variability of the optic radiation in postmortem specimens. In a landmark study of the optic radiation in 25 hemispheres, the key finding was that of anatomic variability between subjects (Ebeling & Reulen, 1988). The distance from the temporal pole to Meyer's loop varied from 22 to 37 mm (mean 27 mm), whereas Meyer's loop was up to 10 mm in front or 5 mm behind the temporal horn. A safety zone of 10 mm anterior to the tip of the temporal horn was thus proposed. Subsequent studies confirm these findings (Table S1) and have been used to postulate different surgical approaches to the temporal lobe via the Sylvian fissure to avoid the optic radiation (Choi et al., 2006), but these remain to be validated in clinical practice.

## Diffusion Tensor Imaging Tractography of the Optic Radiation

### Approaches to tractography

Determining the location of the optic radiation to assist surgery is helpful for several reasons. The optic radiation shows high anatomic variability (Ebeling & Reulen, 1988), cannot be delineated on conventional MRI sequences, and cannot be visually identified during surgery. Diffusion tensor imaging tractography is an MRI technique that enables the depiction of white matter tracts such as the optic radiation in vivo (Duncan, 2010). For example, it can depict the three separate parts of the optic radiation in healthy volunteers (Yamamoto et al., 2005).

Initially *deterministic* algorithms were employed, which are simple and fast to implement but sensitive to effects of noise and do not model regions of crossing fibers or high curvature such as Meyer's loop well. When deterministic tractography was used in healthy controls to measure the distance from the temporal pole to Meyer's loop (TP-ML), a range of 34–51 mm (mean 44 mm) was found and Meyer's loop appeared posterior to the temporal horn in all cases, which does not agree with dissectional studies (Nilsson et al., 2007). Other studies have confirmed that deterministic algorithms fail to delineate the most anterior portions of Meyer's loop, the main region of interest for temporal lobe epilepsy surgery (Table S2).

The alternative is *probabilistic* algorithms that model the noise and uncertainty in the data and are more robust but require lengthy processing. These techniques give results comparable to dissection studies with Meyer's loop more anterior (24–34 mm, mean 28 mm) than previous deterministic studies (Sherbondy et al., 2008). A direct comparison of deterministic and probabilistic approaches in 11 controls and 7 patients gave a TP-ML distance of 32–51 mm (mean 41 mm) for the deterministic algorithm and 17–42 mm (mean 30 mm) for the probabilistic algorithm (Nilsson et al., 2010). It would seem advisable to use probabilistic algorithms for temporal lobe surgery.

**Figure 2:**
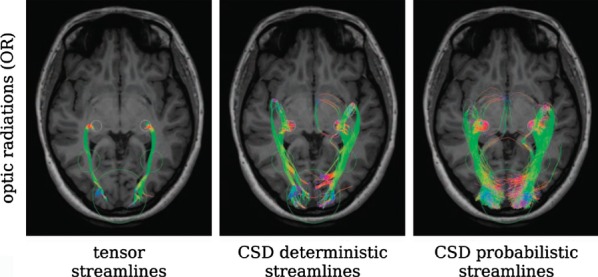
Depiction of the optic radiation using tractography based on the diffusion tensor with deterministic tractography (left), constrained spherical deconvolution with deterministic tractography (middle), or probabilistic tractography (right). Reproduced with permission from Tournier et al. (2012).

The limitations of the diffusion tensor model in dealing with crossing or fanning fibers may be addressed by higher order models such as constrained spherical deconvolution (Tournier et al., 2007). This is superior to diffusion tensor tractography in depicting the fanning fibers of the corticospinal tract (Farquharson et al., 2013) and gives an excellent depiction of the optic radiation (Fig. 2; Tournier et al., 2012). Its use is currently being assessed for planning epilepsy surgery.

### Correlation with visual outcome

In two patients undergoing ATLR, preoperative tractography superimposed on postoperative imaging showed disruption of the optic radiation in the patient with a VFD, but an intact Meyer's loop in the unaffected patient (Powell et al., 2005). Preoperative tractography measurements were related to outcome in patients undergoing ATLR or SAH (Taoka et al., 2008). In those without a postoperative VFD, Meyer's loop was on average 5.0 mm behind the resection margin, whereas in those developing a complete quadrantanopia the resection involved on average 7.5 mm of Meyer's loop. For patients undergoing ATLR, both TP-ML distance and resection size are predictive of the degree of postoperative VFD, with the former having a greater effect (Yogarajah et al., 2009).

### Surgical planning

**Figure 3:**
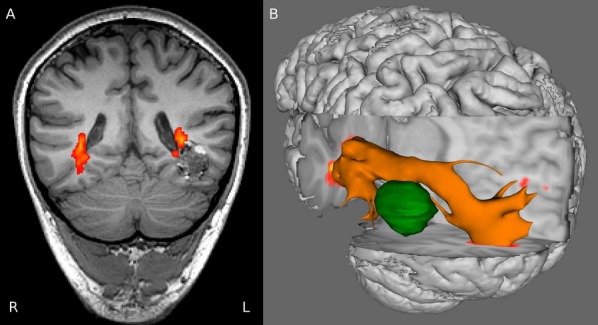
Coronal image showing optic radiation passing superomedial over cavernoma (A). Three-dimensional rendering viewed from the left clearly shows the displacement of the tract by the cavernoma (B). Reproduced with permission from Winston et al. (2011b).

Tractography data may assist surgical planning with preoperative tractography of the corticospinal tract, arcuate fasciculus, or optic radiation altering surgical plans in 80% of patients undergoing tumor surgery (Romano et al., 2007). Likewise, preoperative visualization of the optic radiation has been shown to help the surgeon to plan both temporal and extratemporal epilepsy surgery (Fig. 3; Winston et al., 2011b). However, it is important to recognize that tractography results may vary according to the method employed so need to be tailored to the type of surgery (Winston et al., 2011a). Careful attention to the seed regions may improve results (Benjamin et al., 2012), and as probabilistic tractography is time consuming for the operator, automation to reduce the observer variability may be beneficial (Clatworthy et al., 2010).

### Surgical guidance

The logical next step is to use these data during surgery for intraoperative guidance. The two main approaches are frameless stereotactic neuronavigation, which is widely available, and intraoperative MRI, of which there are relatively few installed systems due to the expense.

### Frameless stereotactic neuronavigation

**Figure 4:**
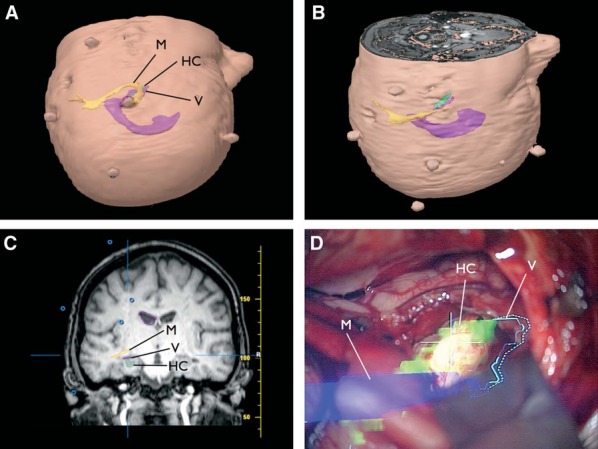
Simulated surgeon's view mimicking a transsylvian approach (A) or a potentially safer subtemporal approach (B). Coronal view showing Meyer's loop overlying the temporal horn (C). Intraoperative view after ventricle entry with image injection on the head-up display of the navigation-bound operating microscope (D). HC, hippocampus; M, Meyer loop; V, ventricle. Reproduced with permission from Thudium et al. (2010). Promotional and commercial use of the material in print, digital or mobile device format is prohibited without the permission from the publisher Lippincott Williams & Wilkins. Please contact journalpermissions@lww.com for further information.

This employs the principle of stereotaxy in which fiducial markers are combined with optical (or other) sensors to detect the position of surgical instruments in relation to the preoperative imaging, most commonly anatomic imaging, but tractography data may also be incorporated (Stone & Rutka, 2008). For example, preoperative tractography of Meyer's loop superimposed on the head-up surgical display was used to guide entry into the temporal horn in patients undergoing transcortical or subtemporal SAH (Thudium et al., 2010) (Fig. 4). On entering the temporal horn, cerebrospinal fluid (CSF) leakage led to unacceptable brain shift so image guidance was no longer possible and the inability to update preoperative imaging in response to brain shift is a major limitation. Nevertheless, neuronavigation may be combined with other modalities such as visual evoked potentials (Kamada et al., 2005).

### Intraoperative MRI

**Figure 5:**
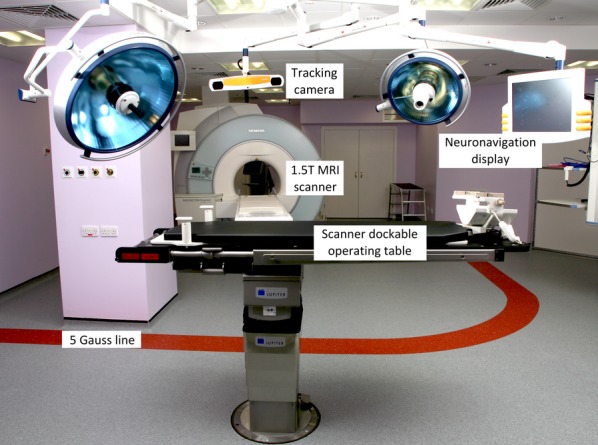
Intraoperative MRI setup at National Hospital for Neurology and Neurosurgery, Queen Square, London. Copyright 2013 UCL ION/UCLH NHNN/Medical Illustration.

Because significant brain shift occurs during surgery (Nabavi et al., 2001), intraoperative imaging may be used to compensate. Fluoroscopy, ultrasound, and computed tomography have been employed (Jolesz, 1995), but MRI is particularly attractive due to its relatively high spatial and temporal resolution, excellent soft tissue contrast, directly acquired multiplanar scans for planning surgical trajectories, and absence of radiation exposure. A typical setup is shown in Fig. 5. In this setting, surgery is performed outside the 5 Gauss line with conventional surgical instruments, but the patient can be transferred to the scanner to acquire updated images during surgery.

Intraoperative MRI was first used in epilepsy surgery to ensure complete resection (Buchfelder et al., 2000, 2002), since incomplete resection is the most common cause of surgical failure (Wyler et al., 1989). However, more recently, tractography data have been incorporated. In a large cohort of patients undergoing ATLR, preoperative and intraoperative imaging including tractography was used to show that the amount of intraoperative damage to Meyer's loop was predictive of the postoperative VFD (Chen et al., 2009).

This study also demonstrated significant brain shift between the preoperative and intraoperative tractography (horizontal up to 11.1 mm, vertical up to 7.8 mm) so that preoperative tractography would no longer be valid without compensation. Although tractography can be performed on intraoperatively acquired scans, limitations imposed by data quality and the available processing time mean that only deterministic algorithms are possible, resulting in a poor depiction of Meyer's loop. In some cases, damage in this region prevents the successful delineation of Meyer's loop. Nevertheless, display of preoperative tractography without correction for brain shift was of benefit in a recent series of patients undergoing extratemporal lobe resection for epilepsy (Sommer et al., 2013), although in only a minority was the optic radiation the tract of interest.

A recently proposed approach to compensate for brain shift is to acquire and process tractography preoperatively and then map this on to updated intraoperative imaging using computational techniques that determine the brain shift (Daga et al., 2012). This technique when applied to postoperative imaging accurately explained the degree of postoperative VFDs (Winston et al., 2012). However, whether compensation for brain shift yields additional benefit beyond the display of the optic radiation to the surgeon still remains to be determined with studies underway.

## Conclusions

Temporal lobe surgery is an effective treatment for epilepsy, but it puts Meyer's loop at risk, with the majority of patients experiencing a postoperative VFD. The ability to drive is a key aim for patients undergoing surgery and a proportion do not meet visual criteria for driving following surgery.

Anatomic dissection has characterized the variability in the location of Meyer's loop, whereas epilepsy surgery has yielded information about the organization of the optic radiation and the nature of VFDs. Meyer's loop is typically just anterior to the temporal horn, an important landmark for temporal lobe surgery, and the degree of the characteristically pseudowedge-shaped partial superior quadrantanopia is related to the amount of damage to this structure. Different approaches to surgery, including the modified Spencer temporal lobectomy or selective amygdalo-hippocampectomy, could pose a lesser risk to vision but have not been systematically studied.

Diffusion tensor imaging tractography has the potential to delineate the optic radiation, but more time-consuming probabilistic algorithms are necessary to accurate delineate Meyer's loop. Most studies have concentrated on predicting the visual outcome and preoperative planning. More recently tractography has been used for real-time neurosurgical guidance with neuronavigation and interventional MRI. These techniques show promise to reduce the degree of VFDs and thus increase the number of patients eligible to drive, but they need further assessment.

## Supporting Information

**Table S1**. Klingler's fiber dissection of the optic radiation. **Table S1**. Anatomic variability of the optic radiation by diffusion imaging.

Figure S1 Retinotopic organization of the optic radiation.
