# Supplementary material for: Epilepsy surgery, vision, and driving: What has surgery taught us and could modern imaging reduce the risk of visual deficits?
Source: Epilepsia. 2013 Sep 20;54(11):1877–88. doi: 10.1111/epi.12372 (PMC4030586; doi:10.1111/epi.12372)
Supplement: Supplementary file 1 — Table S1. Klingler's fiber dissection of the optic radiation. Table S1. Anatomic variability of the optic radiation by diffusion imaging. [file epi0054-1877-sd1.docx]

| **Reference** | **Hemispheres** | **TP-ML distance (mean)** | **ML-TH distance (mean)** |
| --- | --- | --- | --- |
| (Ebeling & Reulen 1988) | 25 | 22 to 37mm (27mm) | -5 to 10mm (5mm) |
| (Sincoff et al. 2004) | 10 | Not specified | >0mm in all |
| (Peuskens et al. 2004) | 17 | 15 to 30mm | -4 to 6mm |
| (Rubino et al. 2005) | 20 | 22 to 30mm (25mm) | 1 to 3mm (2mm) |
| (Choi et al. 2006) | 10 | 28 to 34mm (31.4mm) | >0mm in all |
| (Pujari et al. 2008) | 5 | 22 to 30mm (25mm) | Not specified |

Supplementary Table 1. Klingler's fibre dissection of the optic radiation. *TP-ML distance*: temporal pole to Meyer’s loop distance. *ML-TH distance*: Meyer’s loop to temporal horn distance (+ = ML anterior to TH, - = ML posterior to TH)

| **Paper** | **Subjects** | **Tractography method** | **TP-ML distance (mean)** | **ML-TH distance (mean)** | **ML/TH relationship** |
| --- | --- | --- | --- | --- | --- |
| (Yamamoto et al. 2005) | 5 controls | D | 33.1-40.0mm (37.3mm) | -4.3mm to -3.7mm (-4.0mm) | ML posterior |
| (Nilsson et al. 2007) | 7 controls | D | 34-51mm (44mm) | -21mm to -8mm (-16mm) | ML posterior |
| (Taoka et al. 2008) | 14 patients | D | 30.0-43.2mm (36.6mm) |  |  |
| (Sherbondy et al. 2008) | 8 controls | P | 24-34mm (28mm) | -1mm to +8mm (3mm) | ML anterior |
| (Chen et al. 2009) | 48 patients | D | 20.9-51.5mm (32.1mm) |  |  |
| (Yogarajah et al. 2009) | 20 controls | P | 24-47mm (35mm) | -11 to +9mm (0mm) | ML anterior or posterior |
|  | 21 patients |  | 24-43mm (34mm) | -15 to +9mm (0mm) |  |
| (Mori et al. 2009) | 10 controls | D | 26.1-58.0mm (40.3mm) |  |  |
| (Wang et al. 2010) | 16 controls | D | 26.6-48.9mm (36.3mm) (Method 1,Operator A) |  |  |
|  |  | D | 26.8-48.2mm (36.3mm) (Method 1, Operator B) |  |  |
|  |  | D | 20.8-48.4mm (35.9mm)  (Method 2, Operator B) |  |  |
| (Nilsson et al. 2010) | 11 controls & 7 patients | D | 32-51mm (41mm) | -21mm to -8mm (-14mm) | ML posterior |
|  |  | P | 17-42mm (30mm) | -15mm to +10mm (-2mm) | ML anterior or posterior |

Supplementary Table 2. Anatomical variability of the optic radiation by diffusion imaging. *Tractography method*: D = deterministic, P = probabilistic; *TP-ML distance*: temporal pole to Meyer’s loop distance. *ML-TH distance*: Meyer’s loop to temporal horn distance (+ = ML anterior to TH, - = ML posterior to TH).
